# Differential and paradoxical roles of new-generation antidepressants in primary astrocytic inflammation

**DOI:** 10.1186/s12974-021-02097-z

**Published:** 2021-02-18

**Authors:** Jia-Hui He, Rong-Pei Liu, Yi-Man Peng, Qing Guo, Lan-Bing Zhu, Yi-Zhi Lian, Bei-Lei Hu, Hui-Hui Fan, Xiong Zhang, Jian-Hong Zhu

**Affiliations:** 1 Department of Geriatrics & Neurology, the Second Affiliated Hospital and Yuying Children’s Hospital, Wenzhou Medical University, Wenzhou, 325027 Zhejiang China; 2 Department of Preventive Medicine, Wenzhou Medical University, Wenzhou, 325035 Zhejiang China; 3 Department of Neurology, The Second Affiliated Hospital, Zhejiang University Medical College, Hangzhou, 310009 Zhejiang China

**Keywords:** Astrocytes, SSRI, SNRI, Antidepressant, Neuroinflammation

## Abstract

**Background:**

Selective serotonin reuptake inhibitors (SSRIs) and serotonin-norepinephrine reuptake inhibitors (SNRIs) are commonly used new-generation drugs for depression. Depressive symptoms are thought to be closely related to neuroinflammation. In this study, we used up-to-date protocols of culture and stimulation and aimed to understand how astrocytes respond to the antidepressants.

**Methods:**

Primary astrocytes were isolated and cultured using neurobasal-based serum-free medium. The cells were treated with a cytokine mixture comprising complement component 1q, tumor necrosis factor α, and interleukin 1α with or without pretreatments of antidepressants. Cell viability, phenotypes, inflammatory responses, and the underlying mechanisms were analyzed.

**Results:**

All the SSRIs, including paroxetine, fluoxetine, sertraline, citalopram, and fluvoxamine, show a visible cytotoxicity within the range of applied doses, and a paradoxical effect on astrocytic inflammatory responses as manifested by the promotion of inducible nitric oxide synthase (iNOS) and/or nitric oxide (NO) and the inhibition of interleukin 6 (IL-6) and/or interleukin 1β (IL-1β). The SNRI venlafaxine was the least toxic to astrocytes and inhibited the production of IL-6 and IL-1β but with no impact on iNOS and NO. All the drugs had no regulation on the polarization of astrocytic A1 and A2 types. Mechanisms associated with the antidepressants in astrocytic inflammation route via inhibition of JNK1 activation and STAT3 basal activity.

**Conclusions:**

The study demonstrated that the antidepressants possess differential cytotoxicity to astrocytes and function differently, also paradoxically for the SSRIs, to astrocytic inflammation. Our results provide novel pieces into understanding the differential efficacy and tolerability of the antidepressants in treating patients in the context of astrocytes.

**Supplementary Information:**

The online version contains supplementary material available at 10.1186/s12974-021-02097-z.

## Introduction

Selective serotonin reuptake inhibitors (SSRIs) and serotonin-norepinephrine reuptake inhibitors (SNRIs) are commonly used drugs for depression. A meta-analysis of acute treatment of unipolar major depression in adults indicated differential results of efficacy and acceptability of the new-generation antidepressants. Venlafaxine and sertraline were more efficacious than fluoxetine, fluvoxamine, and paroxetine. Citalopram and sertraline showed the best profile of acceptability, leading to significantly fewer discontinuations than fluvoxamine, paroxetine, and venlafaxine [1]. In treatment of the major depressive disorder in children and adolescents, only fluoxetine was more effective than placebo, and better in tolerability [2]. For the generalized anxiety disorder, citalopram and venlafaxine were more efficacious and with relatively better acceptability than sertraline and fluoxetine. Paroxetine was effective but poorly tolerated [3].

A number of studies have shown that depressive symptoms are closely related to inflammation, with evidence including a higher risk of depression in inflammation and diseases comprising inflammatory processes, increased levels of pro-inflammatory markers among depressed individuals, and antidepressant treatment for pro-inflammatory agent-induced depressive symptoms [4–6]. It is thus desired to understand whether SSRIs and SNRIs regulate neuroinflammation in differential capacities and ways, thereby contributing to their respective unique role in relief of depression. Microglia and astrocytes are the main inherent immune cells in the brain that mediate the development of inflammation [7]. Microglia cells upon activation proliferate and secrete cytokines and chemokines to act on surrounding cells, thereby exerting neuroprotective or neurotoxic effects [8]. SSRIs including fluoxetine and paroxetine have been shown to similarly suppress lipopolysaccharide (LPS)-induced microglia activation while the efficacy of SNRI venlafaxine is marginal [9]. A study focusing on paroxetine demonstrates that its amelioration of microglia activation is via differential regulation of mitogen-activated protein kinase (MAPK) signaling [10]. With accumulating evidence from microglia studies, it has been considered that antidepressants may owe a portion of their therapeutic effectiveness to their anti-inflammatory properties [9, 11, 12].

However, it has not been determined how the antidepressants act on astrocytic activation. Astrocytes, the most abundant cells in the central nervous system, participate in various functions such as promoting neuronal development, synapse formation, and forming the blood-brain barrier, and also play a pivotal role in regulating inflammation [13]. When the brain is injured, reactive astrocytes may exhibit two types of activation, A1 type associated with neurotoxicity and A2 type associated with neuroprotective activity [14]. Activated microglia can induce astrocytes transforming to A1 reactive astrocytes, leading to further death of neurons. A1 astrocytes are abundantly expressed in the brain of various human neurodegenerative diseases, including Alzheimer’s disease and Parkinson’s disease [15].

Primary astrocytes are often used to study astrocytic specific responses. The current method of purification and culture requires serum for days to weeks [16]. The contact of quiescent astrocytes with serum can induce irreversible reactive changes in the cells, which however was not present in vivo unless by injury or impairment of blood-brain barrier [17, 18]. Recently, a protocol using chemically defined neurobasal-based serum-free medium is developed, thereby the isolated astrocytes are in physiologically alike quiescent state [19, 20]. Also, a cytokine mixture (CytoM) comprising complement component 1q (C1q), tumor necrosis factor α (TNF-α), and interleukin 1α (IL-1α) has been demonstrated to be able to best mimic in vivo reactive microglia-elicited stimulation [15]. In this study, we applied these up-to-date protocols and aimed to understand how astrocytes respond to SSRIs including paroxetine, fluoxetine, sertraline, citalopram and fluvoxamine, and SNRI venlafaxine in regard with inflammatory responses and the associated mechanisms. Our results may provide further insight in understanding the role of anti-inflammatory properties of antidepressants in their therapeutic effectiveness against depressive symptoms.

## Materials and methods

### Cell culture and reagents

**Fig. 1:**
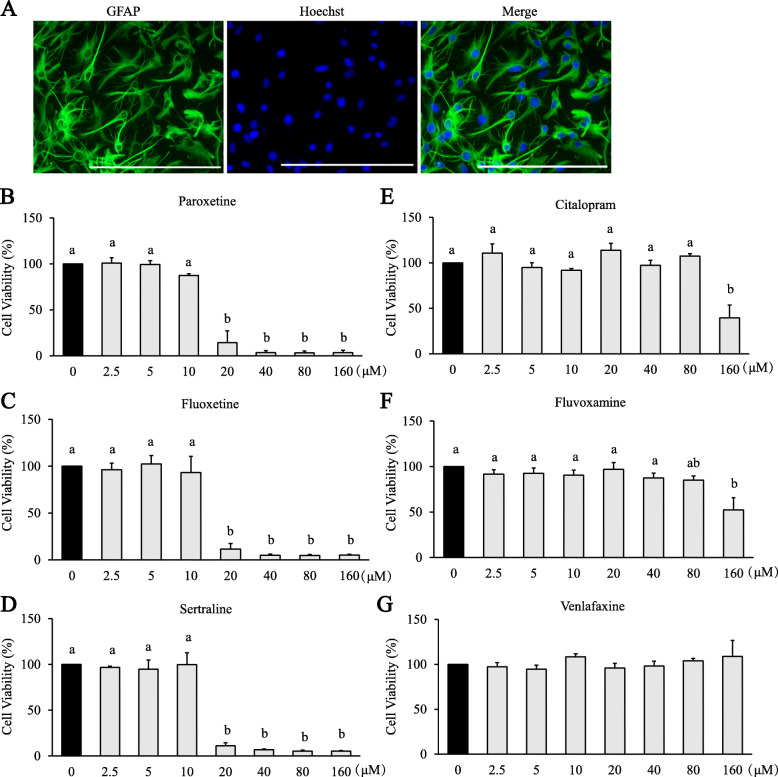
Impact of antidepressants on viability of primary astrocytes. **A** Representative images of isolated primary astrocytes. Green, GFAP; blue, nuclei; bar size, 200 μm. **B**–**G** Cells were treated with paroxetine (**B**), fluoxetine (**C**), sertraline (**D**), citalopram (**E**), fluvoxamine (**F**), and venlafaxine (**G**) at different concentrations for 24 h. Cell viability was expressed as percentage of the control (0 μM), which was set as 100%. Values are means ± SE, *n* = 3. Statistical comparisons were performed using one-way ANOVA, followed by Tukey’s post hoc test. Different letters indicate *p* < 0.05

Primary cultures of astrocytes were prepared from hemispheres of postnatal (day 0 to day 2) Sprague-Dawley rats according to the methods described previously with minor modifications [16, 20]. In brief, the hemispheres were removed from rat pups, cleaned of the meninges and the choroid plexus, and digested with 4 °C dissection medium (10 mM HEPES, 100 U/mL penicillin, and 100 μg/mL streptomycin in Dulbecco’s Hank’s balanced salt solution). Cortices were then cut into small pieces and incubated with 0.05% trypsin-EDTA (diluted in Dulbecco’s phosphate-buffered saline) in a 37 °C water bath for 20 min. The tissue was then washed with cold dissection medium three times and triturated in the DMEM^+^ medium (DMEM supplemented with 10% fetal bovine serum and 100 U/mL penicillin and 100 μg/mL streptomycin). Following a filtration, the supernatant was transferred into a 75-cm^2^ flask pre-coated with poly-l-lysine (1 mg/mL) and cultured at 37 °C. The medium was refreshed after 20–22 h, followed by a refreshing of half of the medium every 3 days. When cells reached a confluency of 90–95%, the flask was placed on an orbital shaker at 250 rpm and 37 °C for 12 h. The remaining attached cells were then collected and re-cultured with a refreshing of half of the medium once a week. Purity of the astrocytes was evaluated by immunostaining of glial fibrillary acidic protein (GFAP) and considered qualified when GFAP positivity was over 95% (Fig. 1A). After reaching confluency, cells were collected, centrifugated, and resuspended in NB^+^ medium (neurobasal medium supplemented with 1× B-27 supplement, 1× Gluta-max, 5 ng/mL HBEGF, 100 U/mL penicillin, and 100 μg/mL streptomycin) for plate seeding. The final concentrations of CytoM components were 30 ng/mL of TNF-α, 3 ng/mL of IL-1α, and 400 ng/mL C1q [15].

HEPES (H1090), the antibiotics (P1400), and poly-l-lysine (P2100) were purchased from Solarbio (Beijing, China). DMEM (C11995500BT), trypsin-EDTA (25200056), neurobasal medium (21103-049), B-27 (17504-044), and Gluta-max (35050-061) were from Gibco (Grand Island, NY, USA). Fetal bovine serum was from Wisent (086-150; Shanghai, China). HBEGF (NBP2-34920), TNF-α (NBP-35185), and IL-1α (NBP-35107) were from Novus (Minneapolis, MN, USA). C1q was from ProSpec (Pro-636; Rehovot, Israel). Inhibitors SP600125 (S1876) and AG490 (HY-12000) were respectively purchased from Beyotime (Shanghai, China) and MedChemExpress (Monmouth Junction, NJ, USA).

### Immunofluorescence

As previously described [21], cells were fixed in 4% paraformaldehyde for 30 min and washed with phosphate-buffered saline (PBS), followed by permeabilization in 0.2% Triton X-100 for 20 min. Samples were blocked with 5% bovine serum albumin (ST023; Beyotime, Shanghai, China) for 1 h and then incubated with primary antibodies against GFAP (MAB360; Merck Millipore, Billerica, MA, USA), p65 (8242; Cell Signaling, Boston, MA, USA), C3 (EPR9394; Abcam, Cambridge, UK), or S100A10 (11250-1-AP; Proteintech, Rosemont, IL, USA) at 4 °C overnight. Following washes with PBS, samples were incubated with Alexa Fluor anti-mouse (A11001) or anti-rabbit IgG (A21428) at room temperature for 1 h, and subsequently incubated with Hoechst 33258 (H3569; all these three from Thermo, Rockford, IL, USA) for 5 min. Coverslips were mounted on glass slides via Fluoromount (P0126; Beyotime, Shanghai, China), and observed with Nikon Eclipse Ti-S.

### Western blot analysis

Cells were lysed in sample buffer containing 60 mM Tri-HCl, pH 6.8, 2% SDS, and 5% glycerol, and boiled for 10 min. Total protein concentration was measured using a BCA kit (P0010; Beyotime, Shanghai, China). Equal amount of protein from each sample was loaded and analyzed by Western blot as previously described [22]. Primary antibodies against p-STAT3 (9145), STAT3 (12640), p-JNK1/2 (9251), JNK1/2 (9252), p-p38 (9211), p38 (9212), p-ERK1/2 (9101), ERK1/2 (9102), inducible nitric oxide synthase (iNOS; 2977) and β-actin (4970), and anti-rabbit secondary antibody (7074) were all purchased from Cell Signaling (Boston, MA, USA). The West Dura Extended Duration substrate detection kit was from Thermo (34076; Rockford, IL, USA).

### Cell viability measurement

Cell viability was determined by the tetrazolium salt 3-[4,5-dimethylthiazol-2-yl] -2,5-diphenyltetrazolium bromide (MTT; Sigma, St. Louis, MO, USA) assay [23]. Primary astrocytes were seeded in triplicates in 24-well plates at 1 × 10^5^ per well and cultured for 7 days in the NB^+^ medium. Astrocytes were treated with different antidepressants at different concentrations for 24 h following overnight starvation. Thereafter, MTT was added into each well and incubated at 37 °C for 1 h. The resulting formazan crystals were dissolved in dimethyl sulfoxide (Sigma, St. Louis, MO, USA). Optical density was measured at 490 nm.

### NO production assay

Medium nitrite was measured as an indicator of NO production [24]. In brief, 50 μl of supernatant was mixed with an equal volume of Griess reagent I, followed by an addition of 50 μl of Griess reagent II (Beyotime, Shanghai, China) at room temperature. Absorbance was immediately measured at 540 nm. The concentration of each sample was calculated from a standard curve generated using sodium nitrite.

### Determination of cytokine production

Medium IL-1β and IL-6 were measured using ELISA kits from R&D Systems (DY501-05 and DY506-05; Minneapolis, MN, USA) according to the manufacturer’s instructions. Goat serum was from Gibco (16210-064; Grand Island, NY, USA). Absorbance was read at 450 nm and 540 nm, respectively. The concentration of each sample was calculated from the standard curve prepared using the included standards.

### Statistical analysis

Data were analyzed by one-way analysis of variance (ANOVA) followed by Tukey’s post hoc test for multiple comparisons and two-way ANOVA for factorial design experiments, using the SPSS 18.0 statistics software. Values were expressed as mean ± SE from at least three independent experiments. *P* < 0.05 was considered statistically significant.

## Results

### Impact of antidepressants on viability of primary astrocytes

Isolated primary astrocytes (Fig. 1A) were treated with different concentrations of antidepressants from 0 to 160 μM. Data from the MTT exclusion test indicated that after 24 h paroxetine, fluoxetine and sertraline led to apparent reduction in cell viability starting at 20 μM [paroxetine: *F*_(7,16)_ = 78.743, *p* < 0.001; post hoc (only the first listed if there were two or more significant values, e.g.), *p* < 0.001 (20 μM vs 0 μM); sertraline: *F*_(7,16)_ = 40.654, *p* < 0.001; post hoc, e.g., *p* < 0.001 (20 μM vs 0 μM); fluoxetine: *F*_(7,16)_ = 72.540, *p* < 0.001; post hoc, e.g. *p* < 0.001 (20 μM vs 0 μM); Fig. 1B–D]. Treatment of astrocytes with citalopram and fluvoxamine resulted in a reduction in cell viability at 160 μM [fluvoxamine: *F*_(7, 16)_ = 4.631, *p* = 0.005; post hoc, *p* = 0.003 (160 μM vs 0 μM); citalopram: *F*_(7, 16)_ = 8.371, *p* < 0.001; post hoc, *p* = 0.002 (160 μM vs 0 μM); Fig. 1 E and F]. No significant change in cell viability was observed with the treatments of venlafaxine (Fig. 1G). Non-toxic dosages were selected for the following experiments.

### Effects of antidepressants on the CytoM-induced iNOS expression and NO production in primary astrocytes

**Fig. 2:**
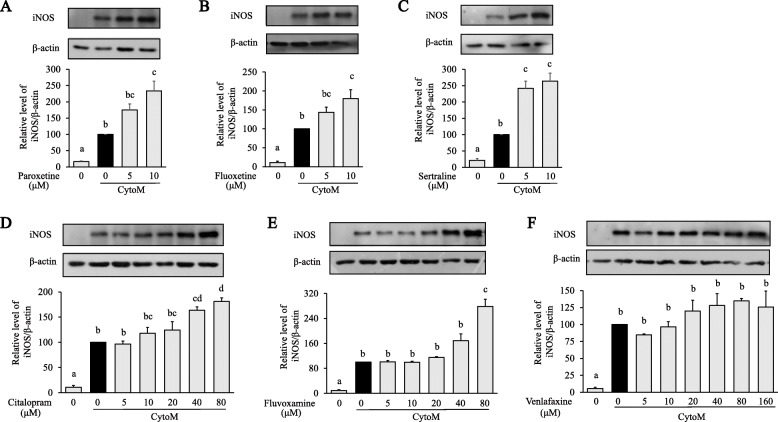
Effects of antidepressants on CytoM-induced iNOS expression in primary astrocytes. Cells were pretreated with paroxetine (**A**), fluoxetine (**B**), sertraline (**C**), citalopram (**D**), fluvoxamine (E) and venlafaxine (**F**) at different concentrations for 30 min followed by stimulation with CytoM for 24 h. Protein levels were quantified and normalized to their respective β-actin levels. Values were expressed relative to the one treated with CytoM alone, which was set as 100. Values are means ± SE, *n* = 3 for (**A**, **C**–**F**), *n* = 4 for (**B**). Statistical comparisons were performed using one-way ANOVA, followed by Tukey’s post hoc test. Different letters indicate *p* < 0.05. iNOS, inducible nitric oxide synthase

The antidepressants alone did not result in iNOS induction in primary astrocytes (data now shown). CytoM treatment induced significant expression of iNOS (Fig. 2). The expression was further increased when cells were pretreated with paroxetine, fluoxetine, and sertraline compared to the CytoM alone group [paroxetine: *F*_(3, 8)_ = 28.426, *p* < 0.001; post hoc, *p* = 0.003 (CytoM + 10 μM vs CytoM); fluoxetine: *F*_(3, 8)_ = 28.737, *p* < 0.001, post hoc, *p* = 0.013 (CytoM + 10 μM vs CytoM); sertraline: *F*_(3, 12)_ = 36.813, *p* < 0.001, post hoc, e.g., *p* = 0.001 (CytoM + 5 μM vs CytoM); Fig. 2A–C; full blots are all in supplemental Figure 1]. Treatment of citalopram or fluvoxamine led to a gradual upward trend of iNOS expression with significant changes being observed at higher concentrations [citalopram: *F*_(6, 14)_ = 26.437, *p* < 0.001; post hoc, e.g., *p* = 0.012 (CytoM + 40 μM vs CytoM); fluvoxamine: *F*_(6, 14)_ = 31.368, *p* < 0.001; post hoc, *p* < 0.001 (CytoM + 80 μM vs CytoM); Fig. 2D and E]. Venlafaxine had no significant effect on iNOS expression (Fig. 2F).

**Fig. 3:**
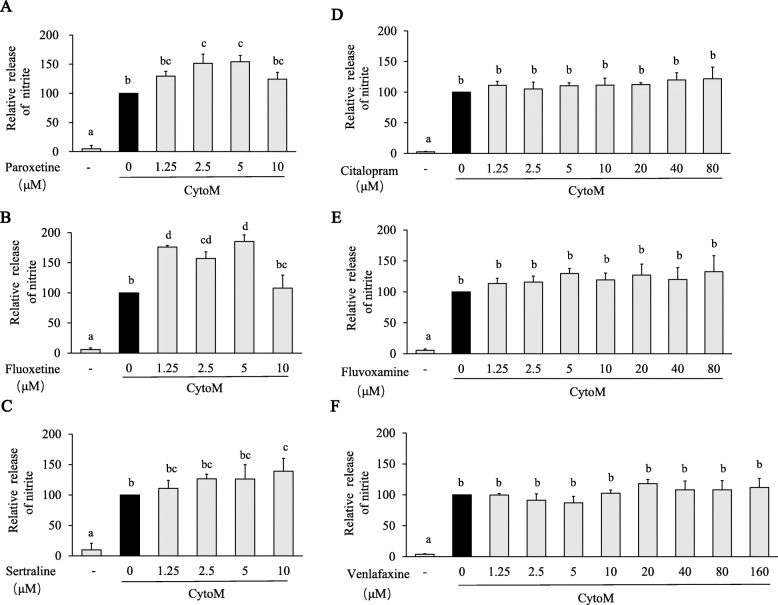
Effects of antidepressants on CytoM-induced NO in primary astrocytes. Cells were pretreated with paroxetine (**A**), fluoxetine (**B**), sertraline (**C**), citalopram (**D**), fluvoxamine (**E**), and venlafaxine (**F**) at different concentrations for 30 min followed by stimulation with CytoM for 24 h. NO production was indicated by nitrite levels in culture media. Values were expressed relative to the one treated with CytoM alone, which was set as 100. Values are means ± SE, *n* = 4 for (**A**, **C**–**E**), *n* = 3 for (**B**, **F**). Statistical comparisons were performed using one-way ANOVA, followed by Tukey’s post hoc test. Different letters indicate *p* < 0.05. NO, nitric oxide

NO production was indicated by measurement of medium nitrite. In line with the iNOS expression, CytoM induced significant production of NO, and the production was further increased by pretreatments with paroxetine, sertraline, and fluoxetine [paroxetine: *F*_(5, 18)_ = 24.005, *p* < 0.001; post hoc, e.g., *p* = 0.005 (CytoM + 2.5 μM vs CytoM); fluoxetine: *F*_(5, 12)_ = 36.773, *p* < 0.001; post hoc, e.g., *p* = 0.004 (CytoM + 1.25 μM vs CytoM); sertraline: *F*_(5,18)_ = 33.797, *p* < 0.001; post hoc, *p* = 0.032 (CytoM + 10 μM vs CytoM); Fig. 3A–C]. Treatments of citalopram, fluvoxamine, and venlafaxine showed no significant effect on the NO production (Fig. 3D–F). The non-elevated nitrite levels with 10 μM of paroxetine and fluoxetine, 40 or 80 μM of citalopram, and fluvoxamine were discordant with the corresponding iNOS expression.

### Effects of antidepressants on the CytoM-induced production of IL-6 and IL-1β in astrocytes

**Fig. 4:**
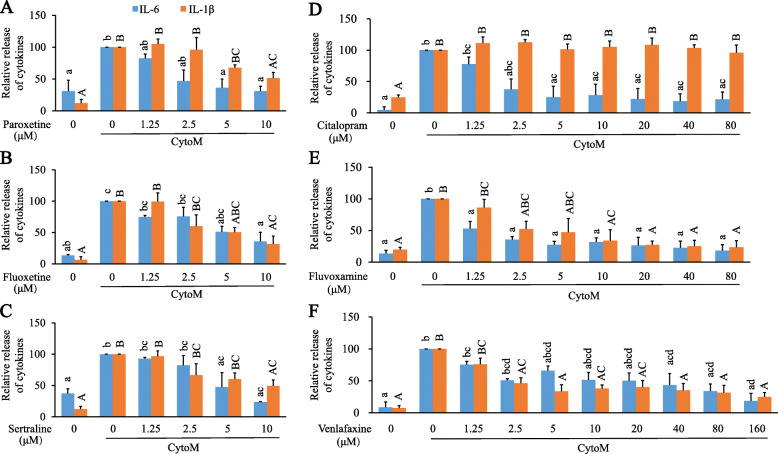
Effects of antidepressants on CytoM-induced IL-6 and IL-1β in primary astrocytes. Cells were pretreated with paroxetine (**A**), fluoxetine (**B**), sertraline (**C**), citalopram (**D**), fluvoxamine (**E**), and venlafaxine (**F**) at different concentrations for 30 min followed by stimulation with CytoM for 24 h. Cytokine levels in culture media were measured. Values were expressed relative to the one treated with CytoM alone, which was set as 100. Values are means ± SE, *n* = 3 for IL-6, *n* = 4 for IL-1β. Statistical comparisons were performed using one-way ANOVA, followed by Tukey’s post hoc test. Different letters within the same cytokine group indicate *p* < 0.05. IL-6, interleukin 6; IL-1β, interleukin 1β

Levels of both IL-6 and IL-1β were significantly elevated upon CytoM stimulation. Different from their impact on iNOS and NO, the six antidepressants all led to inhibition on CytoM-induced IL-6 expression [Fig. 4; paroxetine: *F*_(5, 12)_ = 6.037, *p* = 0.005; post hoc, e.g., *p* = 0.026 (CytoM + 5 μM vs CytoM); fluoxetine: *F*_(5, 12)_ = 11.647, *p* < 0.001; post hoc, e.g., *p* = 0.025 (CytoM + 5 μM vs CytoM); sertraline: *F*_(5, 12)_ = 7.201, *p* = 0.002; post hoc, *p* = 0.007 (CytoM + 10 μM vs CytoM); citalopram: *F*_(8, 18)_ = 5.535, *p* = 0.001; post hoc, e.g., *p* = 0.018 (CytoM + 5 μM vs CytoM); fluvoxamine: *F*_(8, 18)_ = 10.140, *p* < 0.001; post hoc, e.g., *p* = 0.018 (CytoM + 1.25 μM vs CytoM); venlafaxine: *F*_(9, 20)_ = 7.000, *p* < 0.001; post hoc, e.g., *p* = 0.021 (CytoM + 40 μM vs CytoM)].

The CytoM-induced expression of IL-1β was also suppressed by pretreatments of paroxetine, fluoxetine, sertraline, fluvoxamine, and venlafaxine [Fig. 4A–C, E, and F; paroxetine: *F*_(5, 18)_ = 13.822, *p* < 0.001; post hoc, *p* = 0.023 (CytoM + 10 μM vs CytoM); fluoxetine: *F*_(5, 18)_ = 10.938, *p* < 0.001; post hoc, *p* = 0.005 (CytoM + 10 μM vs CytoM); sertraline: *F*_(5, 18)_ = 10.449, *p* < 0.001; post hoc, *p* = 0.023 (CytoM + 10 μM vs CytoM); fluvoxamine: *F*_(8, 27)_ = 5.749, *p* < 0.001; post hoc, e.g., *p* = 0.015 (CytoM + 10 μM vs CytoM); venlafaxine: *F*_(9, 30)_ = 9.792, *p* < 0.001; post hoc, e.g., *p* = 0.03 (CytoM + 2.5 μM vs CytoM)]. Interestingly, citalopram had no effect on the CytoM-induced IL-1β expression (Fig. 4D).

### Effects of antidepressants on the CytoM-induced signaling activation

**Fig. 5:**
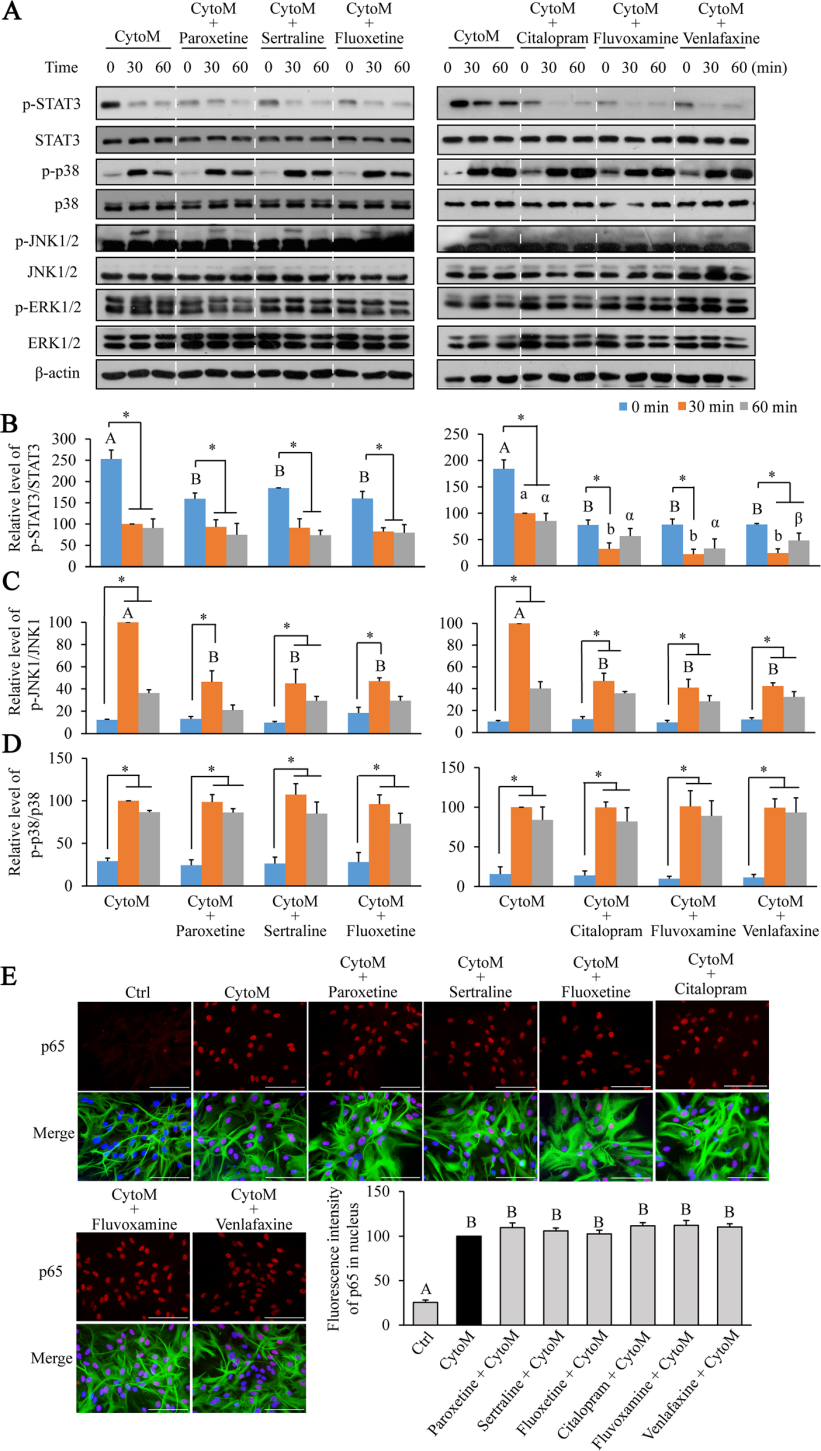
Effects of antidepressants on CytoM-induced signaling activation in primary astrocytes. Cells were pretreated with or without 10 μM of paroxetine, fluoxetine, sertraline, citalopram, fluvoxamine, and venlafaxine for 30 min followed by stimulation of CytoM for 0 to 60 min. **A** Western blot analyses of STAT3, p38, JNK1/2, and ERK1/2 activation. **B**–**D** Levels of p-STAT3 (**B**), p-JNK (**C**), and p-p38 (**D**) were quantified and normalized to their respective total levels. Values were expressed relative to the one stimulated with CytoM alone for 30 min, which was set as 100. Data are means ± SE, *n* = 3. Statistical comparisons were performed using two-way ANOVA. Asterisk between the indicated groups represents *p* < 0.05. Differences within each time point were compared and indicated by one type of letters, that is, English capital for 0 min, English lowercase for 30 min, and Greek lowercase for 60 min. Different letters of the same type represent *p* < 0.05. **E** Representative images and quantification of p65 immunostaining following CytoM stimulation for 30 min. Values were expressed relative to the one stimulated with CytoM alone, which was set as 100. Data are means ± SE, *n* = 3. Statistical comparisons were performed using one-way ANOVA. Different letters indicate *p* < 0.05. Green, GFAP; red, p65; blue, nuclei; bar size, 200 μm

To understand the underlying mechanisms of the differential effects of the antidepressants on CytoM-induced inflammatory responses, we analyzed the MAPKs, STAT3, and NFκB signaling pathways (Fig. 5). CytoM treatment activated p38, JNK1, and p65/NFκB, inhibited STAT3, but had no effect on JNK2 and ERK1/2 in primary astrocytes (Fig. 5 A and E). Pretreatments of the antidepressants did not affect the CytoM-induced activation of p38 and p65 (Fig. 5 D and E). In contrast, the antidepressants per se blunted the baseline STAT3 activity [Fig. 5B; left panel: time, *F*_(2, 24)_ = 50.821, *p* < 0.001; antidepressant, *F*_(3, 24)_ = 3.769, *p* = 0.024; right panel: time, *F*_(2, 24)_ = 28.562, *p* < 0.001; antidepressant, *F*_(3, 24)_ = 28.534, *p* < 0.001]. Amidst, citalopram, fluvoxamine, and venlafaxine further reduced the STAT3 phosphorylation in addition to the CytoM-induced inhibition [post hoc for 30 min, *p* = 0.013 (CytoM + citalopram vs CytoM, *p* = 0.004 (CytoM + fluvoxamine vs CytoM), *p* = 0.003 (CytoM + venlafaxine vs CytoM); for 60 min, *p* = 0.013 (CytoM + venlafaxine vs CytoM)). Meanwhile, the antidepressants inhibited the CytoM-induced JNK1 activation [Fig. 5C; left panel: time, *F*_(2, 24)_ = 72.213, *p* < 0.001; antidepressant, *F*_(3, 24)_ = 10.481, *p* < 0.001; right panel: time, *F*_(2, 24)_ = 93.802, *p* < 0.001; antidepressant, *F*_(3, 24)_ = 19.089, *p* < 0.001; post doc for 30 min, *p* < 0.001 (CytoM + any antidepressant vs CytoM)].

### Blockage of JNK and STAT3 signaling on the CytoM-induced inflammatory responses

**Fig. 6:**
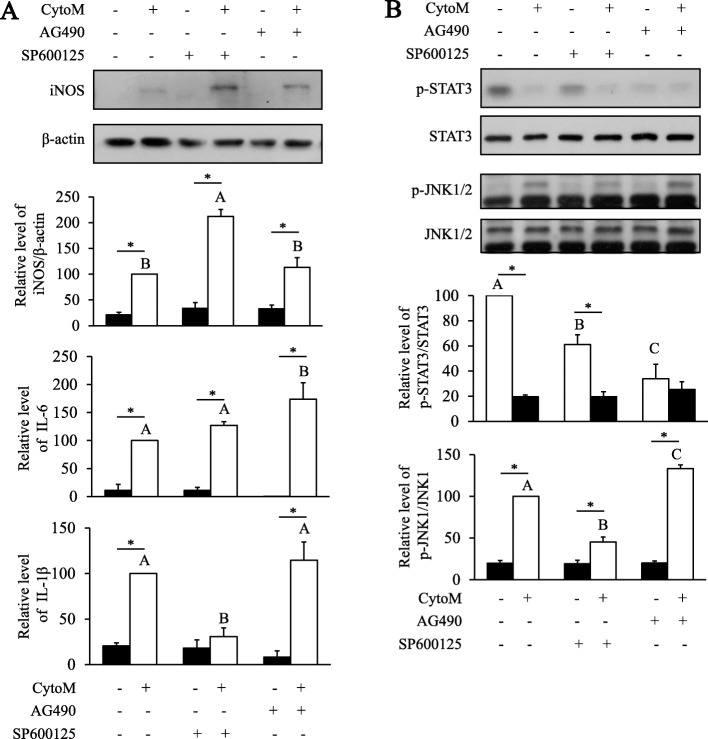
Inhibition of JNK and STAT3 signaling on CytoM-stimulated inflammatory responses in astrocytes. **A** Effects of SP600125 and AG490 on CytoM-induced IL-1β and IL-6 release and iNOS expression. Cells were pretreated with 10 μM of SP600125 or AG490 for 30 min prior to stimulation with CytoM for 24 h. Values were expressed relative to the one stimulated with CytoM alone, which was set as 100. Data are means ± SE, *n* = 3. **B** Effects of SP600125 and AG490 on JNK and STAT3 activation. Cells were pretreated with 10 μM of SP600125 or AG490 for 30 min prior to stimulation with CytoM for 30 min. Levels of p-JNK1 and p-STAT3 were quantified and normalized to the total JNK1 and STAT3 levels. Data are means ± SE, *n* = 4. Statistical comparisons were performed using two-way ANOVA. Different letters and asterisk indicate *p* < 0.05. IL-1β, interleukin 1β; IL-6, interleukin 6

To further demonstrate that the antidepressants may modulate the CytoM-induced inflammatory responses via JNK1 and STAT3 signaling, we used the JNK inhibitor SP600125 and the STAT3 inhibitor AG490. Compared with the group treated with CytoM alone, SP600125 promoted the CytoM-induced expression of iNOS and inhibited the production of IL-1β with no effect on the IL-6 production [Fig. 6A; iNOS: inhibitor, *F*_(2, 12)_ = 17.362, *p* < 0.001; post hoc, *p* < 0.001 (CytoM + SP600125 vs CytoM); IL-1β: inhibitor, *F*_(2, 12)_ = 8.734, *p* = 0.005; post hoc, *p* < 0.001 (CytoM + SP600125 vs CytoM)]. AG490 did not change the expression of iNOS and IL-1β but elevated the CytoM-induced production of IL-6 [IL-6: inhibitor, *F*_(2, 12)_ = 2.891, *p* = 0.094; post hoc, *p* = 0.002 (CytoM + AG490 vs CytoM)]. Effects of the inhibitors on kinase activity were confirmed as in Fig. 6B. Interestingly, results also showed that the baseline STAT3 phosphorylation was inhibited by the JNK inhibitor SP600125 [inhibitor, *F*_(2, 18)_ = 8.945, *p* = 0.002; post hoc, *p* = 0.001], while the CytoM-induced JNK phosphorylation was promoted by the STAT3 inhibitor AG490 [inhibitor, *F*_(2, 18)_ = 68.066, *p* < 0.001; post hoc, *p* < 0.001].

### Antidepressants show no effect on the astrocyte phenotype polarization

**Fig. 7:**
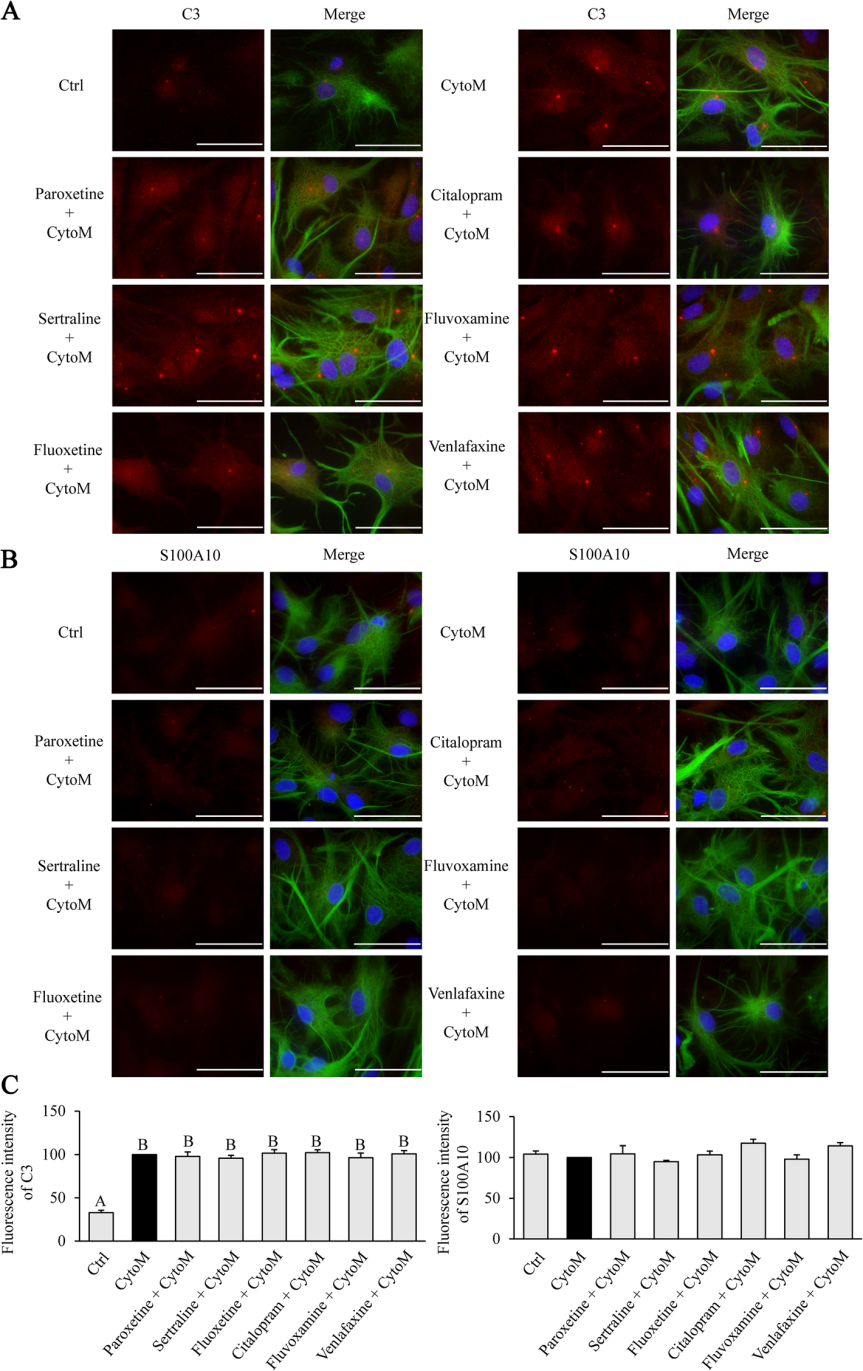
Effects of antidepressants on A1 and A2 phenotype polarization of astrocytes. Cells were pretreated with or without 10 μM of paroxetine, fluoxetine, sertraline, citalopram, fluvoxamine, or venlafaxine for 30 min followed by stimulation with CytoM for 24 h. **A** Representative images of C3 immunostaining. **B** Representative images of S100A10 immunostaining. **C** Quantification of C3 levels (**A**) and S100A10 levels (**B**). Values were expressed relative to the one stimulated with CytoM alone, which was set as 100. Data are means ± SE, *n* = 3. Statistical comparisons were performed using one-way ANOVA. Different letters indicate *p* < 0.05. Green, GFAP; red, C3 or S100A10; blue, nuclei; bar size, 50 μm. C3, complement component 3, indicating A1 type of astrocytes; GFAP, glial fibrillary acidic protein; S100A10, S100 calcium-binding protein-A10, indicating A2 type of astrocytes

It has been reported that the CytoM as composed of C1q, TNF-α, and IL-1α is necessary and sufficient to induce A1 astrocytes [15]. Indeed, the treatment of CytoM led to a conversion of astrocytes from resting (non-reactive) to A1 type as indicated by the elevated expression of C3 (Fig. 7A). In contrast, the expression of S100A10 was not changed by the CytoM treatment (Fig. 7B), suggesting no conversion of the resting cells to A2 type. However, none of the six antidepressants displayed an effect on the expression of C3 or S100A10 (Fig. 7). We also investigated effects of the JNK and STAT3 inhibitors, SP600125 and AG490, on the phenotype polarization of astrocytes. Similarly, no change was observed in the expression of C3 and S100A10 with treatment of either inhibitor (supplemental Figure 2).

## Discussion

**Fig. 8:**
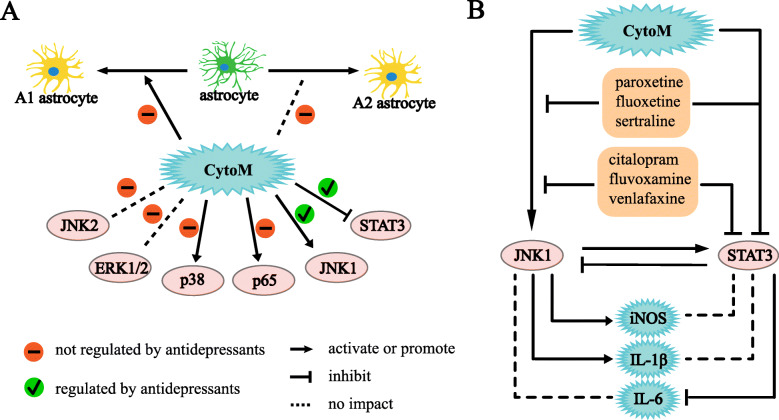
Schematic illustration of antidepressants on CytoM-induced signaling activation and inflammatory responses in primary astrocytes. **a** CytoM activates p38, p65, and JNK1; inhibits STAT3; induces A1 polarization; and has no impact on JNK2, ERK1/2, and A2 polarization. The antidepressants act on JNK1 and STAT3, but not on others. **b** All of the antidepressants inhibit CytoM-induced JNK1 activation. Citalopram, fluvoxamine, and venlafaxine route separately from CytoM to inhibit STAT3 activity. Paroxetine, fluoxetine, and sertraline share the pathways with CytoM to inhibit STAT3 activity. The JNK1 and STAT3 signaling mediate expression of iNOS and IL-1β and expression of IL-6, respectively, and are also cross-talked. IL-1β, interleukin 1β; IL-6, interleukin 6; iNOS, inducible nitric oxide synthase

Neuroinflammation is closely associated with the pathophysiology of various neurological disorders, including primary and secondary depression, Parkinson’s disease, and Alzheimer’s disease [25, 26]. New-generation antidepressants, SSRIs and SNRIs, are wildly used in treatment of depressive symptoms, whereas the mechanisms remain to be further deciphered in regard with how they function and why they display differential efficacies. In this study, we focus on their impacts on astrocytic responses using up-to-date culture and stimulation techniques and demonstrate that the antidepressants differ in cellular toxicity to astrocytes and differentially modulate astrocytic inflammatory responses coupled with unique mechanisms involving JNK1 and STAT3 signaling, while none of the drugs contribute to the A1 and A2 phenotype polarization of astrocytes (Fig. 8).

The SSRIs and SNRIs are designed to specifically impede the activity of neurotransmitter transporters. However, mounting evidence has suggested the existence of various additional targets for these neurotransmitter re-uptake inhibitors, which either support the therapeutic effect or on the contrary trigger adverse events [27–30]. Examples include the protection of paroxetine against dyskinesia in Huntingtin mutant mice and nigrostriatal neurodegeneration in Parkinson’s disease mouse model [31, 32] and the venlafaxine-mediated improvement of cognitive impairment and depressive behavior in multiple sclerosis mouse model [33]. Clinical studies have also found that long-term use of SSRIs may delay conversion from mild cognitive impairment to Alzheimer’s dementia in individuals with previous depression [34], and citalopram and sertraline treatment can improve anxiety symptoms of patients with generalized anxiety disorder [35]. These benefits are observed with an amelioration of inflammation. There is also direct evidence showing inhibition of the antidepressants on NLRP3 inflammasome and lipopolysaccharide-induced microglia activation [9, 10, 36]. While immune cells indeed appear to be the targets for the drugs to take effect, our results warn that the effect can be a double-edged sword. Except for the SNRI venlafaxine, all the SSRIs show a visible cytotoxicity within the range of applied doses, and a paradoxical effect on astrocytic inflammatory responses as manifested by the promotion of iNOS and/or NO and the inhibition of IL-6 and/or IL-1β.

Among the six drugs, venlafaxine has the lowest toxicity to astrocytes, second by citalopram and fluvoxamine, while paroxetine, fluoxetine, and sertraline rank the highest cellular toxicity. It has been reported that paroxetine and fluoxetine induce astrocyte apoptosis by promoting calcium influx and mitochondrial damage [37], and citalopram but not venlafaxine leads to astrocyte death by inducing autophagy [38]. The recommended blood concentration of paroxetine is 0.19–0.32 μM, of sertraline is 0.03–0.15 μM, of fluoxetine is 0.35–0.87 μM, of citalopram is 0.07–0.32 μM, of fluvoxamine is 0.35–0.69 μM, and of venlafaxine is 0.62–1.27 μM. The distribution of psychotropic drugs in the brain is 10 to 40 times more than that in serum [39]. For example, by using fluorine-19 nuclear magnetic resonance spectroscopy, the fluvoxamine steady-state brain concentration is shown at around 12 μM [40], and the fluoxetine concentration achieved in the brain is at 25.5 μM [41]. Thus, the dosages used in the current study are approximately in the range of those exposing to the human brain. Noteworthy, these molecules at higher concentration may interact with some unspecific targets including amine receptors, histamine, adrenergic, and dopaminergic receptors [42].

Roles of the antidepressants in astrocytic inflammation diverge but also converge (Fig. 8). All the SSRIs promote the CytoM-induced expression of iNOS and potentially the production of NO which is indicated by the medium nitrite level. The slight discordance between iNOS and nitrite may attribute to a further oxidation of nitrite to nitrate and the reaction of NO with other free radicals such as superoxide [43–45]. The SNRI venlafaxine does not show such an effect on promotion of iNOS and NO. On the other hand, all the antidepressants inhibit the production of IL-6 with fluvoxamine the best efficiency and venlafaxine the least. All the drugs except citalopram inhibit the production of IL-1β with venlafaxine the best efficiency. This inhibition is in line with a previous report showing that antidepressants induced a reduction in inflammasome activation by an inhibition of IL-1β and IL-18 [36]. As a note, IL-1 molecules stimulate their own and each other’s production, such that IL-1α can significantly induce the expression of IL-1β in alveolar macrophages [46]. This represents an important amplification loop of the inflammatory response [47]. Besides, IL-1α may compete immuno-stimulation induced by IL-1β by interacting with IL-1 receptors [48], while some actions of IL-1β in the brain may be independent of IL-1 receptor 1 [49]. Our measurement of IL-1β is more of a consequence following the IL-1α-containing CytoM stimulation. In addition, the results of IL-1β are mostly comparable to those of IL-6 except for citalopram. Therefore, potential impact of IL-1α on reactivity to IL-1β presumably does not affect the antidepressants in regulation of the IL-1β production. It remains unclear why citalopram has no role in IL-1β mediation. A speculation is that citalopram lacks the specific binding site for interaction with certain IL-1β-regulating molecules. Future investigation may be deserved to understand this interesting observation of citalopram.

Overall, it appears that venlafaxine is the best in astrocytic tolerability and preventing astrocytic inflammation, and fluvoxamine tops over the other SSRIs. As noted earlier, venlafaxine and sertraline are more efficacious, and citalopram and sertraline are with better acceptability in treating major depression in adults [1], but only fluoxetine was effective and better tolerable in children and adolescents [2]. Citalopram and venlafaxine are more efficacious and with better acceptability in treating generalized anxiety disorder [3]. From this point, the effects of the antidepressants on astrocytic cellular toxicity and inflammation do not correlate well with their acceptability and efficacy in treating patients. Indeed, involvement of additional factors such as in neurons and microglia cells should also been taken into account. For example, the antidepressants behave quite differently in microglia in terms of cytotoxicity and anti-inflammation, where paroxetine, fluoxetine, and sertraline protrude as better options [9].

Both common and unique pathways are associated with how the antidepressants regulate inflammatory responses in astrocytes. CytoM induces the cells polarizing to A1 type; activates p38, p65 and JNK1; inhibits STAT3 activity; but does not elicit A2 conversion as well as ERK1/2 and JNK2 activation (Fig. 8B). All the six antidepressants display no impact on the phenotype polarization and the activities or activations of ERK1/2, p38, and p65. In contrast, all the drugs are involved in regulation of the JNK1 and STAT3 signaling, including a consensus inhibition of CytoM-induced JNK1 activation and STAT3 basal activity. As a comparison, in a recent study where primary astrocytes were cultured with serum-containing medium, paroxetine was shown to suppress microglia-conditioned medium-stimulated astrocytic inflammation partially via the p65/NFκB pathway, but not by STAT3 and JNK1/2. In addition, paroxetine showed no impact on lipopolysaccharide-induced astrocytic responses [21]. These results indicate that the phenotype (A1, A2, resting, or mixed) and the stimulus matter how antidepressants play a role in astrocytes.

Noteworthy, mechanisms appear to be different for the antidepressants blocking STAT3 activity. Part of the drugs, including citalopram, fluvoxamine, and venlafaxine, further reduce STAT3 activity on basis of the CytoM-induced reduction, while the remaining part, including paroxetine, sertraline, and fluoxetine, do not. This distinction suggests that citalopram, fluvoxamine, and venlafaxine route separately from CytoM to inhibit STAT3 activity in astrocytes, while the other three may share pathways with CytoM. Further analysis shows that the promotion of iNOS expression and the inhibition of IL-1β production by the antidepressants are mediated by JNK1, but not by STAT3. On the other hand, blockage of the IL-6 production is via STAT3, but not JNK1. Interestingly, a cross talk is observed between JNK1 and STAT3 signaling in astrocytes (Fig. 8B).

## Conclusions

Our results demonstrate that the antidepressants have differential cytotoxicity to astrocytes and function differently, also paradoxically for the SSRIs, to astrocytic inflammation. The mechanisms route via JNK1 and STAT3 signaling. Venlafaxine appears to be the top choice in this context, second by fluvoxamine. Our results provide novel and important pieces into understanding the differential efficacy and tolerability of the antidepressants in treating patients in the context of astrocytes.

## Supplementary Information

**Additional file 1: Supplementary Figure 1.** Full blots for the Figures 1 (A), 3 (B) and 4 (C)

**Additional file 2: Supplementary Figure 2.** Inhibition of JNK and STAT3 signaling on A1 and A2 phenotype polarization of astrocytes

## Data Availability

All data supporting the conclusions of this study are included within the article.

## Abbreviations

C1q: Complement component 1q
C3: Complement component 3
CytoM: Cytokine mixture
GFAP: Glial fibrillary acidic protein
IL-1α: Interleukin 1α
IL-1β: Interleukin 1β
IL-6: Interleukin 6
iNOS: Inducible nitric oxide synthase
MAPK: Mitogen-activated protein kinase
MTT: Tetrazolium salt 3-[4,5-dimethylthiazol-2-yl]-2,5-diphenyltetrazolium bromide
SNRI: Selective serotonin/norepinephrine reuptake inhibitor
SSRI: Selective serotonin reuptake inhibitor
S100A10: S100 calcium-binding protein-A10
TNF-α: Tumor necrosis factor α
