# Supplementary figures and images for: Differential and paradoxical roles of new-generation antidepressants in primary astrocytic inflammation
Source: J Neuroinflammation. 2021 Feb 18;18:47. doi: 10.1186/s12974-021-02097-z (PMC7890881; doi:10.1186/s12974-021-02097-z)

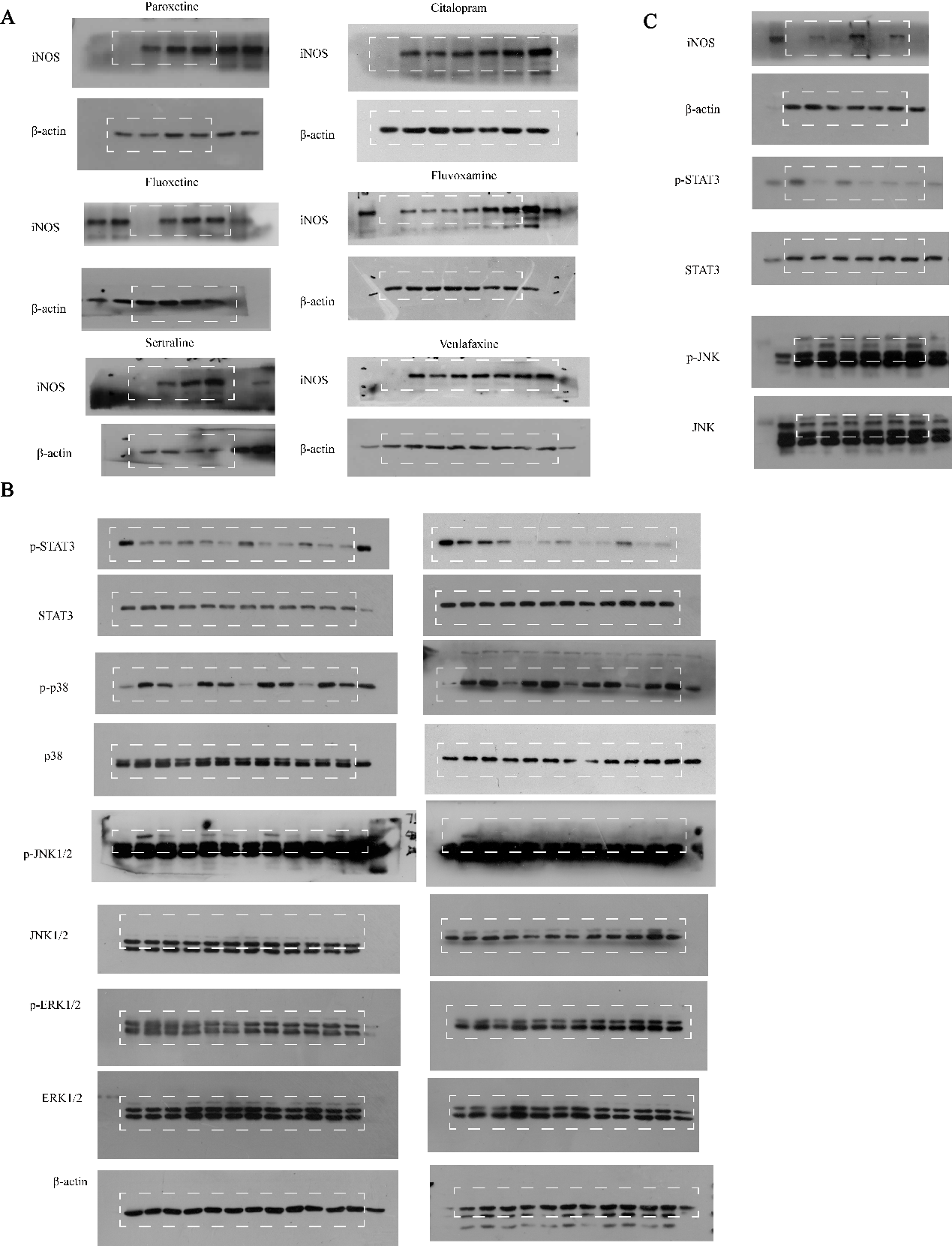


**Supplemental Figure 1.** Full blots for the Figures 1 **(A)**, 3 **(B)** and 4 **(C).**

Supplement: Supplementary file 1 — Additional file 1: Supplementary Figure 1. Full blots for the Figures 1 (A), 3 (B) and 4 (C) [file 12974_2021_2097_MOESM1_ESM.docx]
