# Supplementary material for: Differential and paradoxical roles of new-generation antidepressants in primary astrocytic inflammation
Source: J Neuroinflammation. 2021 Feb 18;18:47. doi: 10.1186/s12974-021-02097-z (PMC7890881; doi:10.1186/s12974-021-02097-z)
Supplement: Supplementary file 2 — Additional file 2: Supplementary Figure 2. Inhibition of JNK and STAT3 signaling on A1 and A2 phenotype polarization of astrocytes [file 12974_2021_2097_MOESM2_ESM.docx]

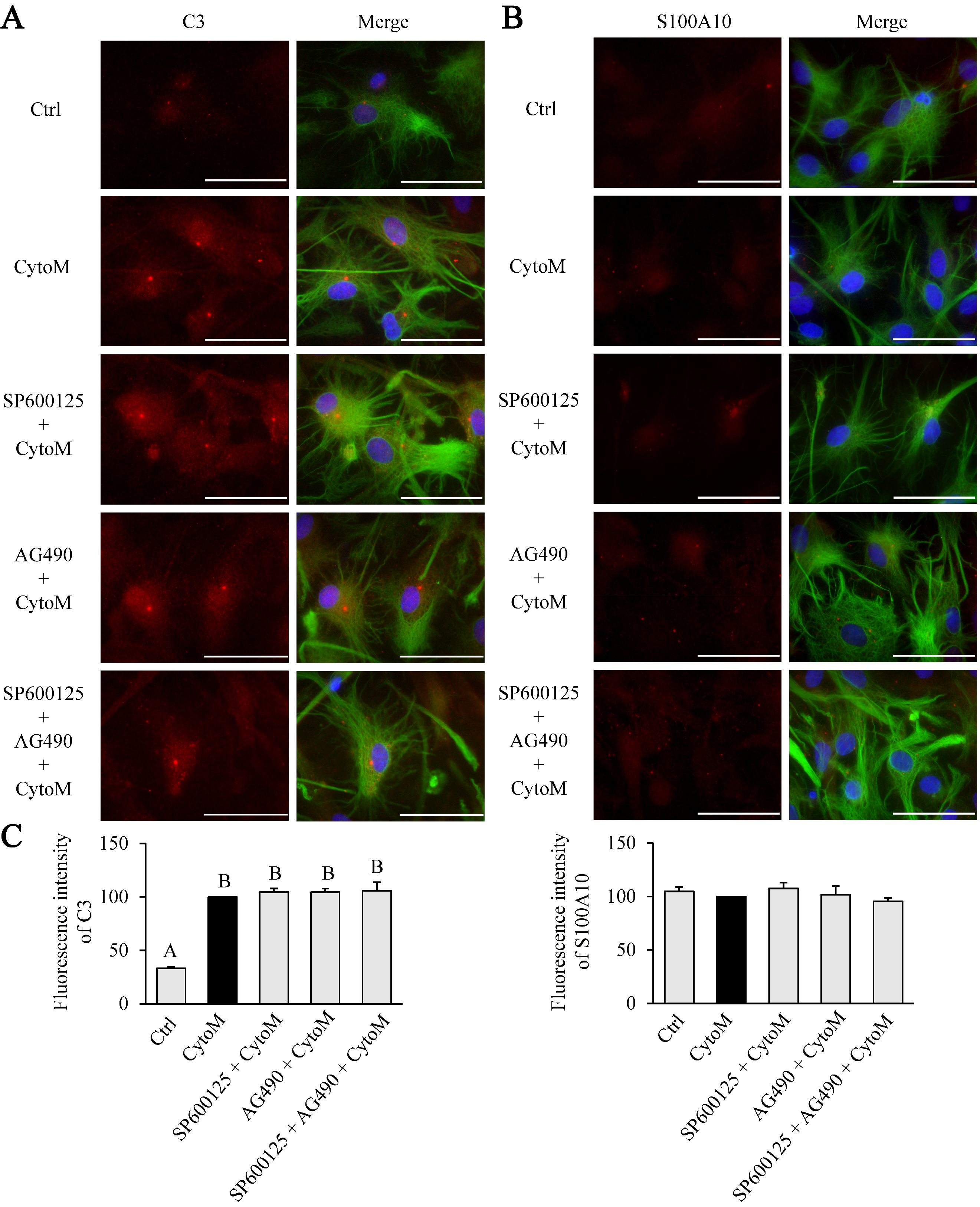


**Supplemental Figure 2. Inhibition of JNK and STAT3 signaling on A1 and A2 phenotype polarization of astrocytes.** Cells were pretreated with or without 10 μM SP600125 or AG490 for 30 min followed by stimulation with CytoM for 24 h. **(A)** Representative images of C3 immunostaining. **(B)** Representative images of S100A10 immunostaining. **(C)** Quantification of C3 levels (A) and S100A10 levels (B). Values were expressed relative to the one stimulated with CytoM alone, which was set as 100. Data are means ± SE, n = 3. Statistical comparisons were performed using one-way ANOVA. Different letters indicate *p* < 0.05. Green, GFAP; red, C3 or S100A10; blue, nuclei; bar size, 50μm. C3, complement component 3; GFAP, glial fibrillary acidic protein; S100A10, S100 calcium-binding protein-A10.
